# MSGD: a manually curated database of genomic, transcriptomic, proteomic and drug information for multiple sclerosis

**DOI:** 10.1093/database/baae037

**Published:** 2024-05-24

**Authors:** Tao Wu, Yaopan Hou, Guanghao Xin, Jingyan Niu, Shanshan Peng, Fanfan Xu, Ying Li, Yuling Chen, Yifangfei Yu, Huixue Zhang, Xiaotong Kong, Yuze Cao, Shangwei Ning, Lihua Wang, Junwei Hao

**Affiliations:** Department of Neurology, Xuanwu Hospital, Capital Medical University, No.45 Changchun Street, Xicheng District, Beijing 100053, China; National Center for Neurological Disorders, No.45 Changchun Street, Xicheng District, Beijing 100053, China; College of Bioinformatics Science and Technology, Harbin Medical University, Baojian Road, Nangang District, Harbin, Heilongjiang 150081, China; Department of Neurology, The Second Affiliated Hospital, Harbin Medical University, Baojian Road, Nangang District, Harbin, Heilongjiang 150081, China; Department of Neurology, The Second Affiliated Hospital, Harbin Medical University, Baojian Road, Nangang District, Harbin, Heilongjiang 150081, China; Department of Neurology, The Second Affiliated Hospital, Harbin Medical University, Baojian Road, Nangang District, Harbin, Heilongjiang 150081, China; Department of Neurology, The Second Affiliated Hospital, Harbin Medical University, Baojian Road, Nangang District, Harbin, Heilongjiang 150081, China; Department of Neurology, The Second Affiliated Hospital, Harbin Medical University, Baojian Road, Nangang District, Harbin, Heilongjiang 150081, China; College of Bioinformatics Science and Technology, Harbin Medical University, Baojian Road, Nangang District, Harbin, Heilongjiang 150081, China; College of Bioinformatics Science and Technology, Harbin Medical University, Baojian Road, Nangang District, Harbin, Heilongjiang 150081, China; Department of Neurology, The Second Affiliated Hospital, Harbin Medical University, Baojian Road, Nangang District, Harbin, Heilongjiang 150081, China; Department of Neurology, The Second Affiliated Hospital, Harbin Medical University, Baojian Road, Nangang District, Harbin, Heilongjiang 150081, China; Department of Neurology, Peking Union Medical College Hospital, Chinese Academy of Medical Sciences and Peking Union Medical College, No.1 Shuaifuyuan, Dongcheng District, Beijing 100730, China; College of Bioinformatics Science and Technology, Harbin Medical University, Baojian Road, Nangang District, Harbin, Heilongjiang 150081, China; Department of Neurology, The Second Affiliated Hospital, Harbin Medical University, Baojian Road, Nangang District, Harbin, Heilongjiang 150081, China; Department of Neurology, Xuanwu Hospital, Capital Medical University, No.45 Changchun Street, Xicheng District, Beijing 100053, China; National Center for Neurological Disorders, No.45 Changchun Street, Xicheng District, Beijing 100053, China

## Abstract

Multiple sclerosis (MS) is the most common inflammatory demyelinating disease of the central nervous system. ‘Omics’ technologies (genomics, transcriptomics, proteomics) and associated drug information have begun reshaping our understanding of multiple sclerosis. However, these data are scattered across numerous references, making them challenging to fully utilize. We manually mined and compiled these data within the Multiple Sclerosis Gene Database (MSGD) database, intending to continue updating it in the future. We screened 5485 publications and constructed the current version of MSGD. MSGD comprises 6255 entries, including 3274 variant entries, 1175 RNA entries, 418 protein entries, 313 knockout entries, 612 drug entries and 463 high-throughput entries. Each entry contains detailed information, such as species, disease type, detailed gene descriptions (such as official gene symbols), and original references. MSGD is freely accessible and provides a user-friendly web interface. Users can easily search for genes of interest, view their expression patterns and detailed information, manage gene sets and submit new MS-gene associations through the platform. The primary principle behind MSGD’s design is to provide an exploratory platform, aiming to minimize filtration and interpretation barriers while ensuring highly accessible presentation of data. This initiative is expected to significantly assist researchers in deciphering gene mechanisms and improving the prevention, diagnosis and treatment of MS.

**Database URL**: http://bio-bigdata.hrbmu.edu.cn/MSGD

## Introduction

Multiple sclerosis (MS) is the most common autoimmune disorder of the central nervous system (CNS) characterized by neuroinflammation and neurodegeneration. In 2020, the Multiple Sclerosis Atlas report stated that, on a global scale, an individual with an average age of 32 is diagnosed with multiple sclerosis every 5 minutes. Currently, approximately 2.8 million people are living with multiple sclerosis worldwide, and this number continues to rise (1). Multiple sclerosis is a complex disease, and in addition to genetic variations, lifestyle and environmental factors also play a significant role in disease risk. The pathophysiology of MS is highly intricate; hence, uncovering precise molecular mechanisms through genomics, transcriptomics, proteomics and related fields represents the foremost challenge.

MS is influenced by both genetic and environmental factors (2–4). First-degree relatives and monozygotic twins of affected individuals have a significantly higher lifetime risk of MS, approximately 7 times and over 100 times higher than that of the general population, respectively, indicating a strong genetic susceptibility to the disease (5–7). Initial research has linked MS susceptibility to the major histocompatibility complex (MHC) locus, paving the way for the identification of other genetic factors (8). Subsequently, a plethora of genetic loci outside the MHC region have been discovered to be associated with the risk of MS. These loci include numerous variant positions in genes such as interleukin-7 receptor (IL7R) (9–12), vitamin D receptor (VDR) (13–16), tumor necrosis factor (TNF) (17–20), interleukin 2 receptor subunit alpha (IL2RA) (21–24) and others.

In recent years, there has been a rapid increase in the number of newly discovered genetic variants. Presently, this information remains highly fragmented, making comprehensive analysis challenging. One adverse consequence of this fragmentation is that efficiently retrieving relevant information from a substantial volume of text has become exceedingly difficult. The establishment of a comprehensive database containing all reliable information related to genetic and clinical data is now considered the optimal approach to meet this need.

To bridge this gap, Multiple Sclerosis Gene Database (MSGD), a manually curated database of experimentally supported gene-MS correlations, has been developed. This database encompasses MS-related gene variations, transcriptomics, proteins and drug-related information. MSGD is expected to serve as a valuable resource for researchers exploring the relationship between genes and MS.

## Methods

### Data collection and management

To ensure the high quality of the database, we referenced the management steps of databases previously established by our team, such as Lnc2Cancer 3.0 (25), LncACTdb 3.0 (26) and NSDNA (27). The key steps for data management are as follows: (i) a PubMed database search was conducted using the following keywords: ‘multiple sclerosis’, ‘experimental autoimmune encephalomyelitis’ and ‘gene’ with the cutoff date set at 20 November 2023, resulting in the retrieval of 5485 published studies related to these topics. (ii) We organized and summarized the relevant information from these studies. Each study was reviewed and analyzed by at least two researchers. (iii) The collected entries were categorized based on the research content into gene variations, mRNA, proteins, gene knockouts/knock-ins, drugs and high-throughput data. (iv) Standardization of gene names was performed, with mouse genes standardized according to Mouse Genome Informatics (http://www.informatics.jax.org/mgihome/nomen/), human genes according to HUGO Gene Nomenclature Committee (https://www.genenames.org) and rat genes according to Rat Genome Database (https://rgd.mcw.edu/nomen/nomen.shtml) and so on. (v) The descriptions of variant were standardized based on the retrieval information from the dbSNP database. This meticulous data collection and management approach ensures the reliability and integrity of the MSGD, making it a valuable resource for researchers exploring the gene–MS relationship.

**Figure 1.**
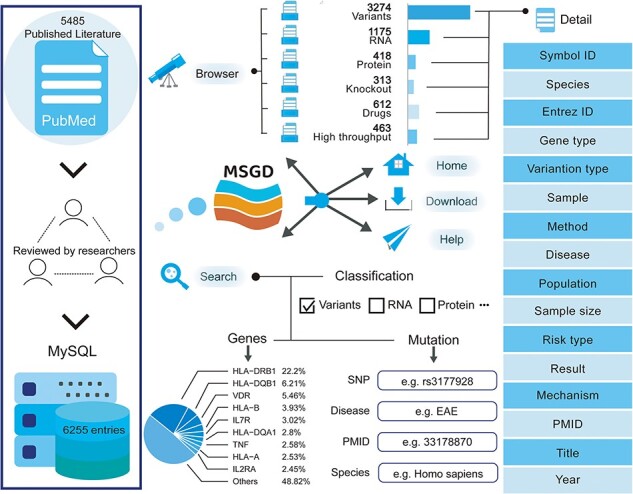
A schematic workflow of MSGD.

We conducted a screening of 5485 publications to compile the current version of MSGD. This database encompasses 6255 entries, comprising 3274 variant entries, 1175 RNA entries, 418 protein entries, 313 knockout entries, 612 drug entries and 463 high-throughput entries. Each entry is comprehensive, detailing species, disease type, specific gene descriptions (such as official gene symbols) and original references (see Figure 1). The database contains 4547 entries based on humans, 1484 entries based on mice, 174 entries based on rats and 50 entries based on other species.

### Database construction

All data in MSGD are stored and managed using MySQL, which is freely accessible data management software (https://www.mysql.com/). The web interface is built using Java Server Pages (https://www.java.com/). The data processing scripts are written in Java, and the web service is hosted on the Apache Tomcat Web server. You can access the MSGD database for free from http://bio-bigdata.hrbmu.edu.cn/MSGD.

## Results

### The web interface for MSGD

**Figure 2.**
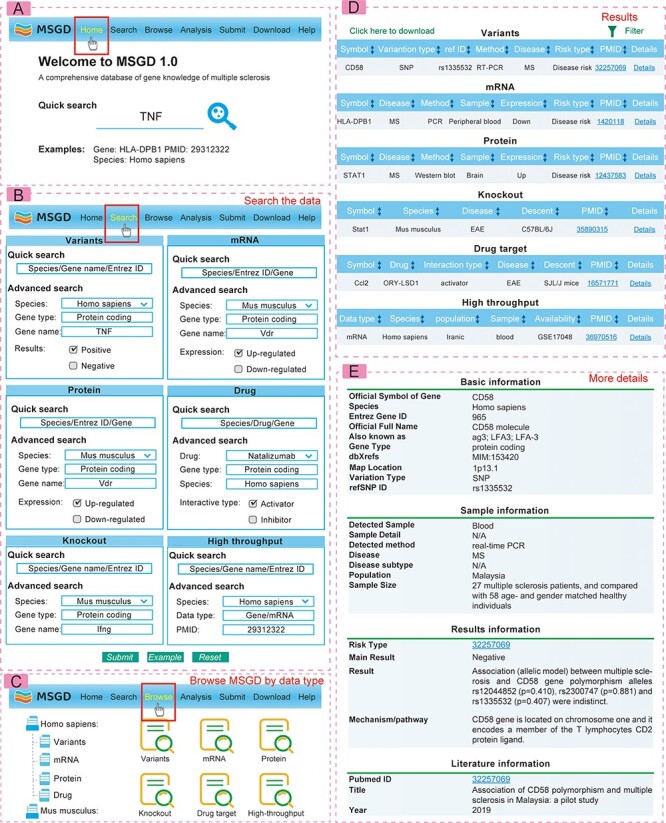
The web interface and usage for MSGD.

The web interface of MSGD is highly user-friendly for database queries (see Figure 2). Users can perform searches based on gene variations and gene symbols on the ‘Search’ page. It’s important to note that MSGD supports fuzzy searching and advanced searching. On the advanced search page for drug targets, users can filter by specific MS subtypes or MS animal models. All possible search results are displayed in tabular form, and users can click on the ‘Detailed Information’ hyperlink to access more specific details from the table. Users can sort the search results by clicking on the column names in ascending or descending order on the search results interface. Additionally, they can perform secondary filtering of results by entering any keyword in the ‘Search’ box. On the ‘details’ page, genes are linked to authoritative annotation databases, while high-throughput data are linked to available database sources.

Users can also explore information based on gene variations, transcriptomics, proteomics, drugs and more on the ‘Browse’ page. We generated ‘Hot Points’ to showcase genes of particular interest in recent publications on multiple sclerosis research, while also considering their search popularity within this database. In the ‘Download’ section, all collected data are available for free download. Furthermore, users have the option to submit new MS–gene association data via the ‘Submit’ page. Submitted data will be included in the database and made available to the public in the next version after review by our submission review committee. For additional guidance, a comprehensive tutorial is provided on the ‘Help’ page.

### Data statistics in MSGD

**Figure 3.**
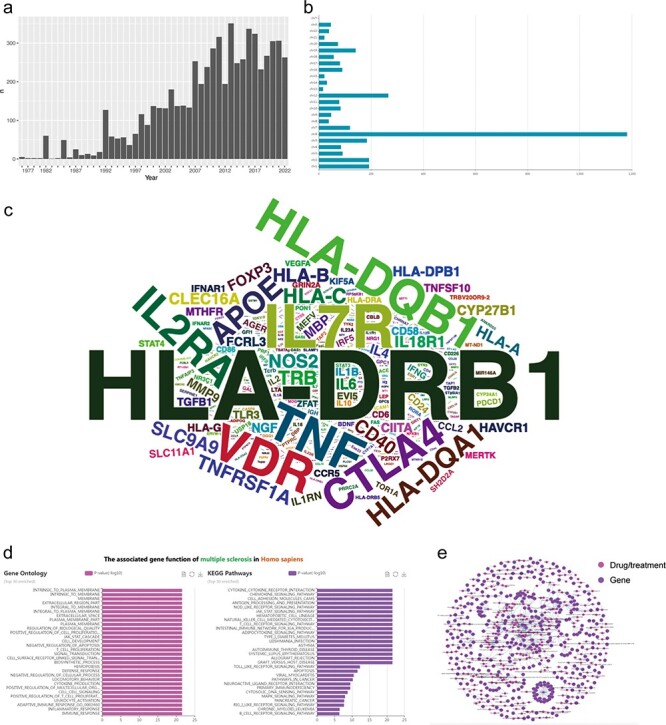
Data statistics, data integration and functional analysis in MSGD. (a) Annual publication counts. (b) Distribution of genetic variants on chromosomes. (c) Word cloud of MS risk genes (the size of the word represents the amount of evidence). (d) GO and KEGG functional enrichment analysis of MS risk genes. (e) MS drug target network. Nodes represent genes or drugs, while edges represent experimentally supported associations between genes and drugs.

We conducted an analysis of the yearly publication count related to MS-associated genes in PubMed (Figure 3a) and observed a noticeable upward trend, particularly in the past decade, signifying a substantial accumulation of research. This suggests an increasing effort by researchers and neurologists to decipher the precise molecular mechanisms involved in MS development. Consequently, genetic research may represent one of the prominent areas of focus in the field of MS over the past decade.

### Data integration and functional analysis

In the analysis section of MSGD, we could find that evidence for MS-related risk genes is widely distributed across the chromosomes, with the highest evidence concentration on chromosome 6 (Figure 3b). Users can click on any chromosome to display the corresponding results. We counted the total amount of evidence for each gene (the top 20 was displayed in ‘Data Statistics’). Furthermore, we analyzed the positive evidence for each gene, generating a word cloud where the size of each word represents the amount of positive evidence for each gene (Figure 3c). According to the positive evidence amount, the top five genes associated with the risk of MS are as follows: HLA-DRB1 (10.76%), IL7R (3.13%), TNF (2.63%), VDR (2.57%) and HLA-DQB1 (2.5%).

Furthermore, MSGD provides the functional enrichment analysis on all genes exhibiting positive correlation results. The Gene Ontology (GO) analysis revealed a significant enrichment of genes associated with the cell membrane. Additionally, the Kyoto Encyclopedia of Genes and Genomes enrichment analysis highlighted their primary involvement in antigen presentation functions and activation of immune cell pathways (Figure 3d). To visualize the complex relationships, we constructed a dual-part network of MS genes and drugs using Cytoscape (version 3.7.1). In this network, nodes represent genes or drugs, while edges represent experimentally supported associations between genes and drugs (Figure 3e).

## Discussion

With the rapid advancements in MS genetics, a substantial volume of genetic and MS-related data has been accumulated (8). However, data on gene-MS associations are scattered throughout various published articles. Therefore, a high-quality database containing comprehensive MS-related gene data is crucial for a thorough understanding of the MS process. Yet, there are currently few databases that provide comprehensive resources for gene–MS associations across different species. Hence, we have developed a specialized MS database known as MSGD, which encompasses a wide range of data, including gene variations, transcripts, proteins, drugs, high-throughput data and more, for various species. This database serves as a valuable resource for researchers looking to explore gene–MS relationships comprehensively.

In addition to collecting a broader range of gene-MS associations, MSGD offers several advantages. Firstly, MSGD provides detailed gene information, including official gene symbols, Entrez Gene IDs, official full names (also known as gene types), map positions and dbXrefs, along with article information as described in the database content. Secondly, MSGD offers cross-species data and a user-friendly web interface for users to retrieve and download all available data. Thirdly, MSGD incorporates data on gene-related variations, targeted drugs and knockout information. Therefore, MSGD is a specialized database that serves as a comprehensive resource for gene–MS associations.

We plan to update the database every 1–2 years, depending on the volume of newly published data during that period. Currently, we are actively collecting relevant data and planning an update to MSGD. The next version will include the following enhancements: Firstly, updates on newly validated gene–MS associations. Secondly, essential interface optimizations based on user feedback. Thirdly, enhanced integration and visualization of high-throughput datasets. Lastly, the incorporation of gene targets for approved drugs or those in clinical trials, alongside RNA expression data. This ongoing effort ensures that MSGD remains an up-to-date and valuable resource for researchers and scientists exploring gene–MS associations in the context of multiple sclerosis.

## Conclusion

In summary, with the support of experimental data, MSGD not only provides a comprehensive specialized database for multiple sclerosis but also offers a broader perspective on gene functions within MS. In the future, we plan to regularly update the database. Furthermore, we have plans to integrate more sources and information, along with providing tools for predicting gene–MS associations. We believe that MSGD will serve as a valuable resource, assisting researchers in deciphering gene mechanisms and improving the diagnosis and treatment of multiple sclerosis.

## Data Availability

All data used in the analysis can be obtained at http://bio-bigdata.hrbmu.edu.cn/MSGD.
